# Specific gut microbiota alterations and metabolic deficits in Parkinson’s disease: a controlled comparison with functional constipation

**DOI:** 10.3389/fcimb.2026.1767241

**Published:** 2026-05-28

**Authors:** Shuang Yang, Chen Tian, Ding Peng

**Affiliations:** 1 Department of Gastroenterology, Xuanwu Hospital Capital Medical University, Beijing, China; 2 Department of Respiratory and Critical Care Medicine, Beijing Haidian Hospital, Beijing, China

**Keywords:** 16S rRNA sequencing, functional constipation, gut microbiota, gut-brain axis, Parkinson’s disease

## Abstract

**Objective:**

To isolate Parkinson’s disease (PD)-specific gut dysbiosis from motility-related changes, this study compared the fecal microbiota of PD patients with constipation (PDC, n=20) against a functional constipation (FC, n=15) control group.

**Methods:**

High-throughput 16S rRNA gene sequencing and functional prediction were performed on fecal samples. Diversity metrics and taxonomic compositions were analyzed to identify PD-specific signatures.

**Results:**

The PDC group exhibited significantly higher microbial richness than the FC group (P = 0.01). Despite structural similarities driven by the shared constipation phenotype (ANOSIM R = 0.084), PDC was distinguished by specific compositional shifts, including the enrichment of *Paraprevotella*, *Akkermansia*, *unclassified Ruminococcaceae*, and *Campylobacter*. Functionally, the PDC microbiome showed significant deficits in tyrosine and glutathione metabolism pathways (P<0.05), indicating compromised dopamine precursor synthesis and antioxidant defense.

**Conclusion:**

By controlling for colonic transit time, this study identified specific microbial alterations and metabolic deficits in PD independent of constipation. These findings support a “gut-first” etiology linked to specific pathobionts and barrier compromise.

## 1 Introduction

Parkinson’s disease (PD) is a prevalent neurodegenerative disorder characterized by cardinal motor symptoms and significant non-motor features. Among these, gastrointestinal (GI) dysfunction, particularly constipation, affects up to 80% of patients and often precedes motor onset by decades (Schapira et al., 2017; Tan et al., 2022). This phenomenon has directed intense scientific scrutiny toward the “microbiome-gut-brain axis, ” positing that the gut may be the initiation site of PD pathology (Sampson et al., 2016).

Emerging evidence supports the “gut-first” hypothesis, suggesting that gut dysbiosis—characterized by the enrichment of pro-inflammatory pathogens and depletion of beneficial commensals—may trigger enteric inflammation and alpha-synuclein aggregation (Braak et al., 2003; Nishiwaki et al., 2020). Such microbial alterations can compromise the intestinal barrier (“leaky gut”) and facilitate neuroinflammation (Vandeputte et al., 2016; Houser and Tansey, 2017; Romano et al., 2021). Recently, Gu et al. (2025) conducted a comprehensive systematic review and meta-analysis of randomized controlled trials, definitively demonstrating that gut microbiota-targeted therapies—such as specific probiotics, prebiotics, and fecal microbiota transplantation (FMT)—yield substantial efficacy in alleviating refractory gastrointestinal dysfunctions and neuroinflammation in PD patients (Gu et al., 2025).

However, a critical limitation in most existing studies is the comparison of PD patients against healthy controls (HC) (Houser and Tansey, 2017). Since constipation itself significantly alters colonic transit time and shapes the gut ecosystem (Barichella et al., 2019; Yuan et al., 2024), it remains challenging to distinguish whether the observed dysbiosis is intrinsic to PD pathology or merely a secondary consequence of intestinal stasis. Therefore, utilizing Functional Constipation (FC) as a disease control is essential to eliminate this confounding factor (Fu et al., 2022).

To isolate the PD-specific microbial fingerprint, this study compared the fecal microbiota of PD patients with constipation (PDC) against a cohort of patients with functional constipation (FC). By strictly controlling for constipation severity, we aimed to characterize specific microecological signatures—independent of motility issues—to elucidate the potential microbial mechanisms underlying Parkinson’s disease (Varesi et al., 2022).

## 2 Methods

### 2.1 Study design and participants

This study included 35 participants who visited Xuanwu Hospital of Capital Medical University between December 2019 and March 2021. Based on the MDS Clinical Diagnostic Criteria for Parkinson’s Disease (2015) and the Rome IV diagnostic criteria for functional constipation, participants were divided into the Parkinson’s disease with constipation group (PDC, n=20) and the functional constipation group (FC, n=15). This study has been approved by the Ethics Committee of Xuanwu Hospital, Capital Medical University (approval number: [2015]-023-revision-1), and all subjects have signed the informed consent form before participating in the study. The data supporting the findings of this study are available from the corresponding author upon reasonable request. Exclusion criteria included: a history of gastrointestinal surgery, use of antibiotics or probiotics within the past 3 months, and comorbidity with severe systemic diseases such as inflammatory bowel disease (IBD).

### 2.2 Fecal sample collection, DNA extraction, and 16S rRNA sequencing

DNA Extraction and 16S rRNA Sequencing: Fresh fecal samples were collected from participants and stored at -80 °C. Microbial genomic DNA was extracted from 200–500 mg of frozen fecal matter using the E.Z.N.A.™ Mag-Bind Soil DNA Kit (OMEGA Bio-tek, USA) following the manufacturer’s instructions. DNA integrity was verified via agarose gel electrophoresis. The V3-V4 hypervariable regions were amplified using universal primers 341F (5’-CCTACGGGNGGCWGCAG-3’) and 805R (5’-GACTACHVGGGTATCTAATCC-3’). A two-stage PCR strategy was employed: the first stage involved 5 initial cycles at low annealing temperature (45 °C) followed by 20 cycles at 55 °C. Amplicons were purified using magnetic beads, quantified with Qubit 3.0. The constructed libraries were sequenced on the Illumina platform using a paired-end sequencing strategy.

### 2.3 Bioinformatics and downstream microbiome analyses

Raw data underwent quality control, assembly, and filtering using cutadapt, PEAR, and PRINSEQ software to remove low-quality sequences. Usearch (v11.0) was used to remove chimeras, and Operational Taxonomic Units (OTUs) were clustered based on a 97% similarity threshold. Taxonomy annotation was performed for OTUs using the RDP classifier (v2.12) against the Silva 16S rRNA database.

Mothur was utilized to calculate alpha diversity indices (including Chao1, Shannon, etc.). Principal Coordinate Analysis (PCoA) based on Bray-Curtis distance was conducted to evaluate beta diversity. Functional profiles were predicted using PICRUSt (v1.1.4) and summarized at the KEGG Level 3 pathway level. Significant pathways were visualized using a differential scatter plot, a heatmap, and a group-wise mean abundance comparison.

### 2.4 Statistical analysis

Statistical analyses were performed using SPSS 22.0 and R software (v3.6.0). Continuous variables were compared between groups using the independent samples t-test or Wilcoxon rank-sum test, while categorical variables were analyzed using the Chi-square test. Differences in community structure were assessed using ANOSIM. Linear Discriminant Analysis Effect Size (LEfSe) (LDA score > 2.0) was used to identify differentially abundant taxa between groups. A P-value of < 0.05 was considered statistically significant.

## 3 Results

### 3.1 Participant characteristics

The demographic and clinical characteristics of the participants are summarized in **Table 1**. No significant between-group differences were observed in age, sex, BMI, duration of constipation, Wexner score, or smoking status (all P > 0.05), suggesting overall comparability between the PDC and FC groups.

**Table 1:** Demographic and clinical characteristics of the participants.

Characteristic	PDC	FC	P
Age	64.80 ± 6.04	62.93 ± 7.91	0.434
Gender (female)	10(50%)	6(40%)	0.570
BMI	24.91 ± 3.75	23.98 ± 2.97	0.441
Duration of Constipation (years)	11.33 ± 13.20	9.07 ± 7.18	0.554
Wexner Score	12.05 ± 4.65	11.60 ± 4.69	0.779
Smoking	6(30%)	3(20%)	0.517
Duration of PD (years)	6.20 ± 4.81	–	–
H-Y Staging	2.03 ± 0.73	–	–

### 3.2 Sequencing depth and alpha diversity

Following high-throughput sequencing and quality control, clean reads were obtained for all samples. Rarefaction curves plateaued, and Good’s coverage indices exceeded 99.0%, indicating sufficient sequencing depth. Regarding alpha diversity, the microbial community richness was significantly distinct between the two groups. The Chao1 index was significantly higher in the PDC group compared to the FC group (444.84 ± 86.39 vs 334.62 ± 146.62, P = 0.01). Similarly, the ACE index was elevated in the PDC group (442.51 ± 85.88 vs 332.35 ± 141.40, P = 0.01). However, no significant differences were observed in species diversity and evenness, as indicated by the Shannon index (3.18 ± 0.79 vs 2.75 ± 0.92, P = 0.16) and Simpson index (0.14 ± 0.19 vs 0.17 ± 0.16, P = 0.51). These alpha-diversity results are summarized in **Table 2**.

**Table 2:** Alpha-diversity indices in the PDC and FC groups.

Alpha-diversity index	FC	PDC	P
Shannon	2.75 ± 0.92	3.18 ± 0.79	0.16
Chao1	334.62 ± 146.62	444.84 ± 86.39	0.01
Simpson	0.17 ± 0.16	0.14 ± 0.19	0.51
ACE	332.35 ± 141.40	442.51 ± 85.88	0.01

### 3.3 Beta diversity analysis

**Figure 1:**
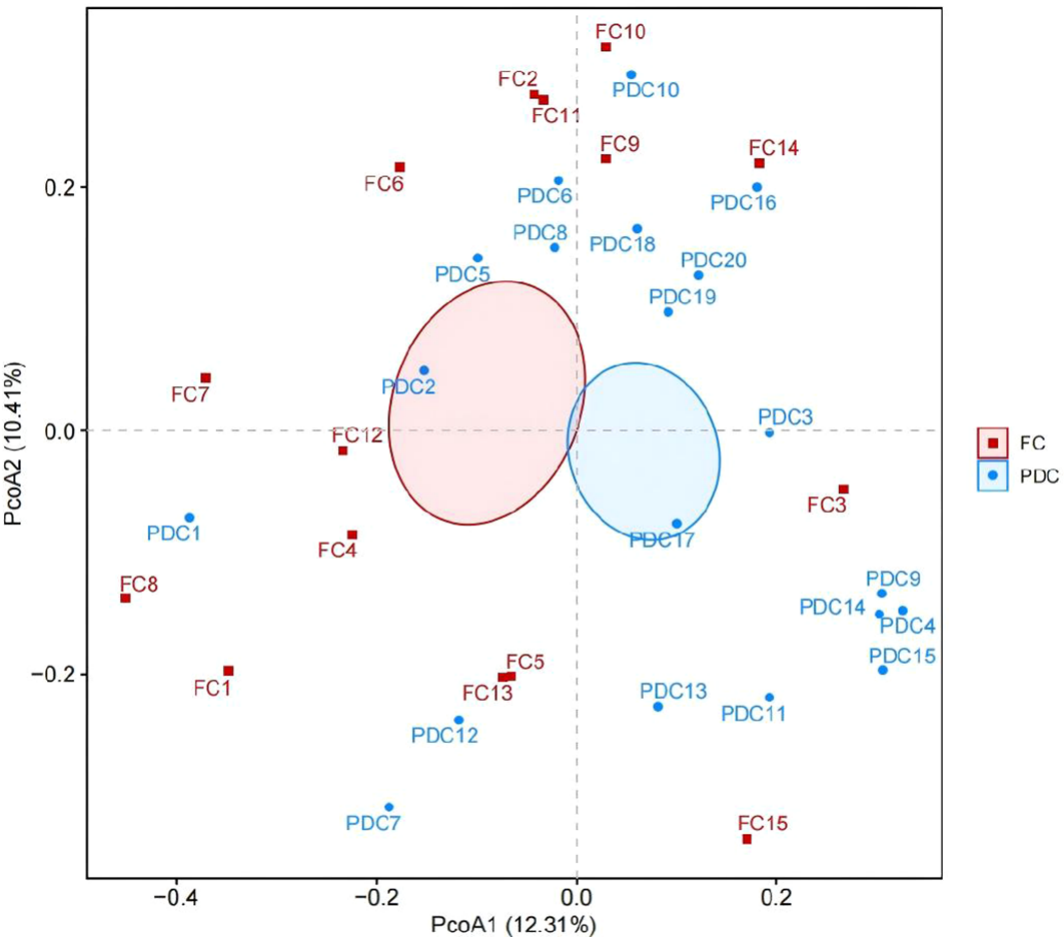
Beta diversity analysis showing a modest but statistically significant separation of gut microbiota structure between the PDC and FC groups. Principal Coordinate Analysis (PCoA) plot based on Bray-Curtis distance. Each point represents an individual sample (red squares represent the FC group; blue circles represent the PDC group). The shaded ellipses represent the 95% confidence intervals for each group, indicating the core distribution of the samples. Statistical significance of the community structure differences between the two groups was assessed using ANOSIM (Analysis of Similarities) (R = 0.084, P = 0.048).

To evaluate the overall differences in microbial community structure, beta diversity analysis was performed. The Principal Coordinate Analysis (PCoA) based on Bray-Curtis distance (**Figure 1**) visualized the structural variation between groups. ANOSIM analysis indicated a statistically significant difference between the two groups (P = 0.048). However, the R value was low (R = 0.084), reflecting considerable structural similarity and overlap between the PDC and FC groups (**Figure 1**).

### 3.4 Taxonomic composition of the gut microbiota

**Figure 2:**
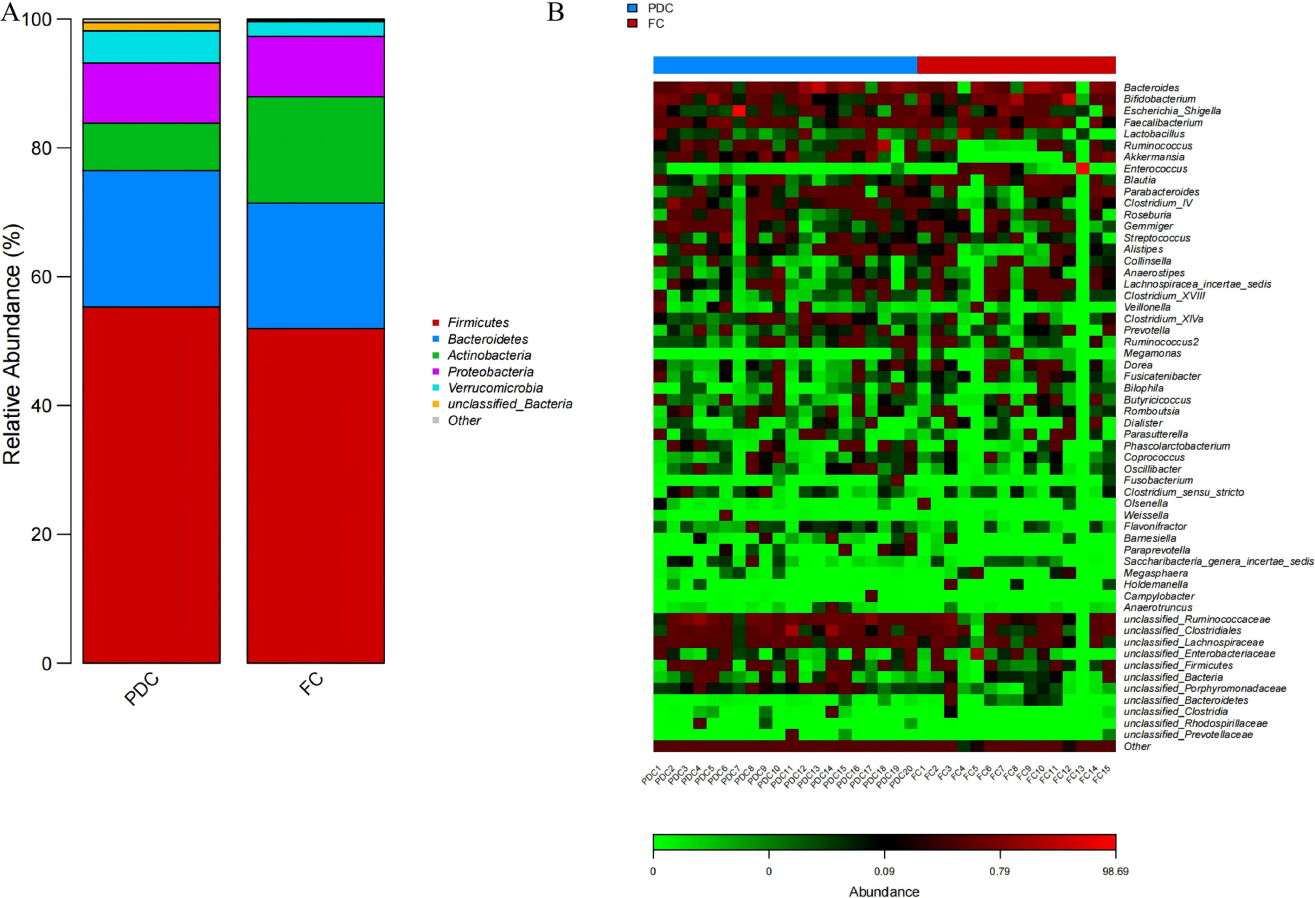
Taxonomic composition of the gut microbiota at the phylum and genus levels. **(A)** Stacked bar chart showing the mean relative abundance of dominant bacterial phyla. Notably, the phylum Verrucomicrobia was significantly enriched in the PDC group compared to the FC group (Wilcoxon rank-sum test, P < 0.05). **(B)** Heatmap illustrating the relative abundance of dominant bacterial genera across all individual samples. The color gradient from green to red indicates low to high relative abundance, respectively.

The overall taxonomic composition at the phylum and genus levels is presented in **Figure 2**. At the phylum level, *Verrucomicrobia* was enriched in the PDC group (**Figure 2A**). At the genus level, the heatmap analysis (**Figure 2B**) highlights the specific bacterial taxa with disparate abundance patterns. The PDC group was characterized by a dramatic expansion of *Paraprevotella*, reaching a mean relative abundance of 17.75% compared to only 0.18% in the FC group (P < 0.01). Additionally, the genus *Akkermansia* showed a significant enrichment in the PDC group (4.64% vs 2.11%, P = 0.004), as did *Clostridium_IV* (2.97% vs 0.59%, P = 0.003).

### 3.5 Differentially abundant taxa identified by LEfSe

**Figure 3:**
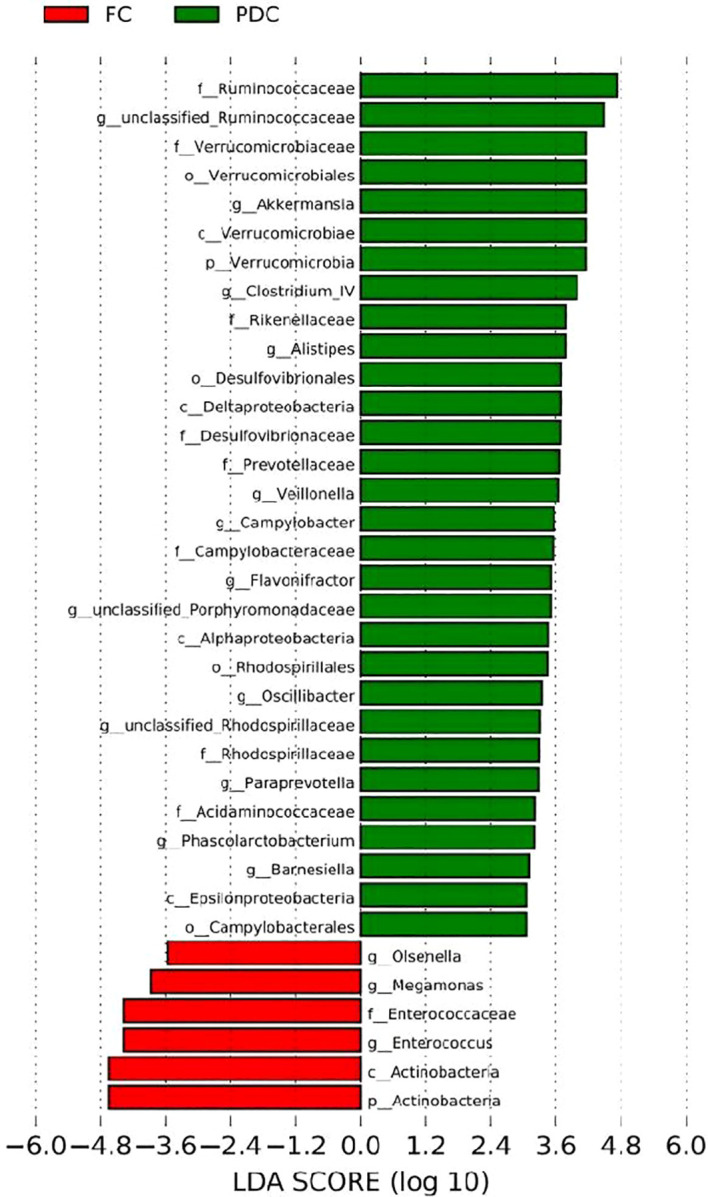
Identification of differentially abundant taxa associated with PDC and FC groups. Linear Discriminant Analysis Effect Size (LEfSe) analysis was performed to identify robust microbial biomarkers. The bar chart displays taxa with an LDA score > 2.0. Red bars indicate bacterial taxa that are significantly enriched in the FC group, while green bars represent taxa significantly enriched in the PDC group. The length of each bar (x-axis) represents the logarithmic LDA score, indicating the effect size of each differentially abundant taxon.

Linear Discriminant Analysis Effect Size (LEfSe) (LDA score > 2.0) was applied to identify robust biomarkers distinguishing the two groups (**Figure 3**). The PDC group was primarily defined by the enrichment of the family Ruminococcaceae (LDA = 4.72) and the genus *unclassified Ruminococcaceae* (LDA = 4.49). Consistent with the abundance data, *Akkermansia* (LDA = 4.15) and *Clostridium_IV* (LDA = 3.99) were also identified as key discriminators for PDC. Notably, despite the high mean abundance of *Paraprevotella*, its LDA score (LDA = 3.28) was moderate, suggesting high inter-individual variability within the PDC group. Furthermore, the analysis identified the potential pathogen *Campylobacter* (LDA = 3.56) as a specific biomarker associated with PDC. Conversely, the FC group was primarily associated with the enrichment of Actinobacteria (LDA = 4.66), *Enterococcus* (LDA = 4.38), and *Megamonas* (LDA = 3.88).

### 3.6 Functional prediction analysis

**Figure 4:**
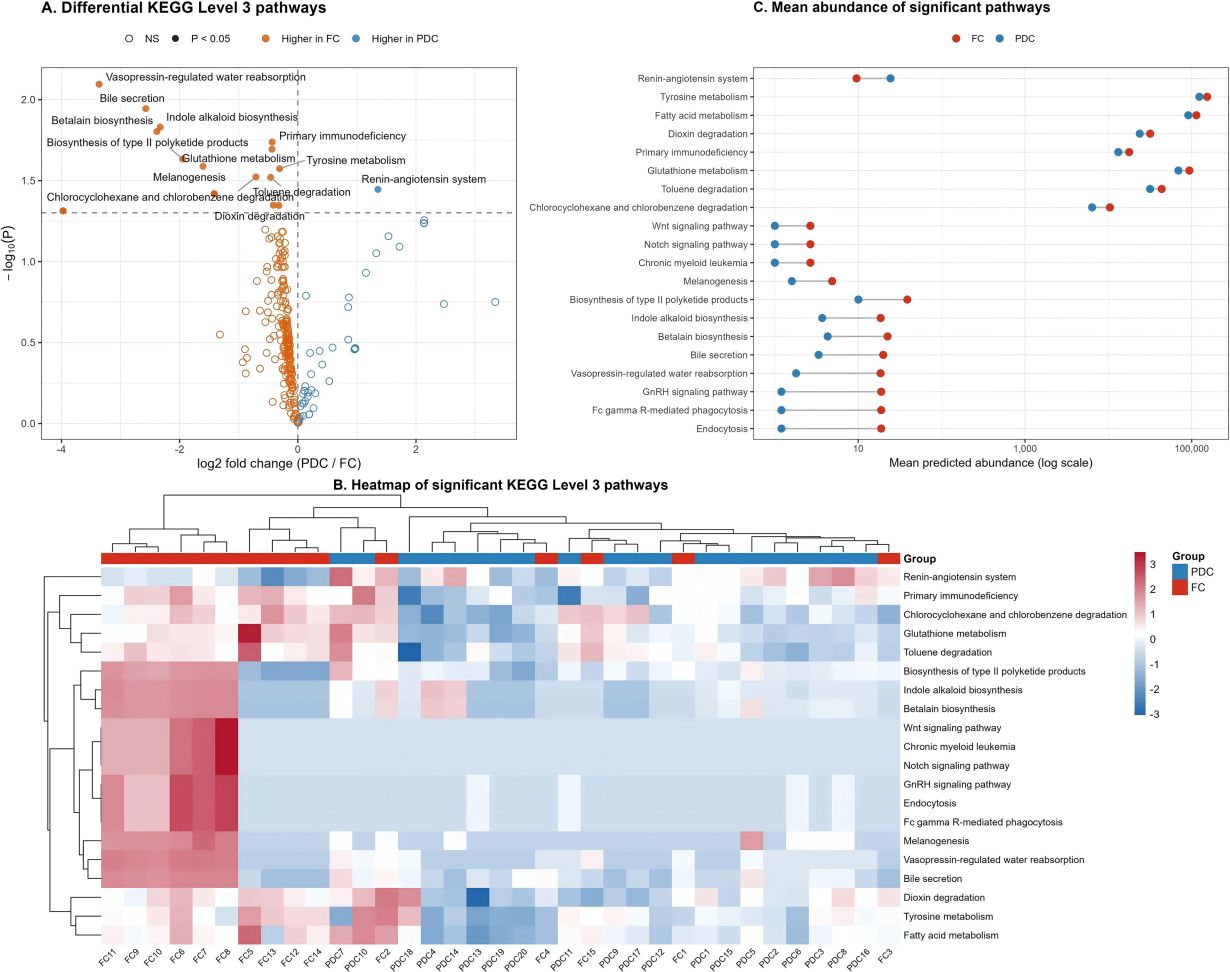
KEGG Level 3 pathway-based functional differences between the PDC and FC groups. **(A)** Scatter plot summarizing differential KEGG Level 3 pathways between groups, with the x-axis indicating log2 fold change (PDC/FC) and the y-axis indicating statistical significance. **(B)** Heatmap of pathways with nominal significance (P < 0.05) across individual samples. Values are log-transformed and row-scaled for visualization. **(C)** Comparison of the mean predicted abundance of nominally altered pathways between the PDC and FC groups. Together, these analyses highlight reduced tyrosine metabolism, glutathione metabolism, toluene degradation, and dioxin degradation in the PDC group.

To explore the functional implications of the observed dysbiosis, we predicted the metabolic potential of the gut microbiota using PICRUSt based on the KEGG database. KEGG Level 3 pathway analysis revealed multiple nominally significant functional differences between the two groups, which are summarized in **Figure 4**. Overall, most significant pathways showed lower predicted abundance in the PDC group than in the FC group, and the heatmap demonstrated a group-related clustering trend at the functional level (**Figures 4A–C**). Among these altered pathways, tyrosine metabolism was significantly depleted in the PDC group compared with the FC group (mean abundance: 123, 393 vs 152, 878, P = 0.027). Glutathione metabolism was also reduced in PDC patients (P = 0.020). In addition, pathways related to environmental xenobiotic degradation, including toluene degradation (P = 0.030) and dioxin degradation (P = 0.045), were decreased in the PDC group. These results suggest that, beyond taxonomic shifts, the PDC microbiome may exhibit broader functional deficiencies involving neurotransmitter precursor metabolism, antioxidant defense, and xenobiotic processing.

## 4 Discussion

By comparing Parkinson’s disease patients with constipation (PDC) to a functional constipation (FC) control group matched for constipation severity, this study identified specific microbial taxa associated with Parkinson’s disease pathology. Our findings reveal that the PDC microbiome is defined by a pathogenic quartet: the enrichment of *unclassified Ruminococcaceae*, the heterogeneous bloom of *Paraprevotella*, the paradoxical increase of mucin-degrading *Akkermansia*, and the emergence of *Campylobacter*. These results suggest that gut dysbiosis in PD involves mechanisms distinct from functional constipation, driven by autoimmunity, metabolic inflammation, and barrier compromise (Houser and Tansey, 2017; Romano et al., 2021; Fang et al., 2021; Zhang et al., 2021).

The beta diversity analysis in this study reveals that, despite the significant overlap in the overall microbiome patterns between PDC and FC largely being constrained by the shared phenotype of “constipation”, the abnormal abundance of specific bacterial taxa, such as *Paraprevotella* and *Akkermansia*, remains a key factor driving significant differences in the pathological characteristics of PD and simple functional constipation. Furthermore, at the functional prediction level, the significant depletion of the tyrosine (a direct precursor of dopamine synthesis) metabolic pathway in the PDC group strongly suggests impaired ability of gut microbiota to synthesize or regulate dopamine precursors; whereas the downregulation of glutathione metabolism reflects weakened intestinal defense against oxidative stress, which may exacerbate the susceptibility of the enteric nervous system to pathogenic factors.

A novel finding of our study is the significant enrichment of the family Ruminococcaceae in the PDC group. This contrasts with several meta-analyses comparing PD patients to healthy controls, which often report a depletion of SCFA-producing bacteria, including Ruminococcaceae (Unger et al., 2016; Shin et al., 2020). However, recent studies focusing on constipation severity have noted that specific genera within Ruminococcaceae (e.g., *Ruminococcus*) can be positively associated with colonic transit time and constipation severity (Roager et al., 2016). Our use of an FC control group suggests that the enrichment of specific *unclassified Ruminococcaceae* taxa might represent a compensatory response to the altered gut environment or a PDC-specific enterotype that differs from the general PD population.

Notably, *Paraprevotella* exhibited the highest mean relative abundance (17.75%) in the PDC group but with a moderate LDA score, indicating substantial inter-individual variability. *Paraprevotella* is a major producer of succinate (Morotomi et al., 2009). While traditionally viewed as a metabolic intermediate, recent evidence identifies succinate as a “danger signal” that activates the succinate receptor (SUCNR1) to trigger type 2 inflammation and macrophage activation (Harber et al., 2020). The overgrowth of *Paraprevotella* in a subset of patients may therefore drive a pro-inflammatory metabolic state, potentially exacerbating systemic inflammation in PD (Hertel et al., 2019).

We identified *Campylobacter* as a specific biomarker for the PDC group. This finding aligns with the molecular mimicry hypothesis seen in Guillain-Barré Syndrome (GBS), where *Campylobacter jejuni* lipooligosaccharides (LOS) mimic human GM1 gangliosides (Godschalk et al., 2004; Yuki and Hartung, 2012). Given that GM1 gangliosides play a critical role in stabilizing lipid rafts and preventing alpha-synuclein aggregation in PD (Schneider et al., 2015), we hypothesize that *Campylobacter*-induced cross-reactive antibodies might disrupt this neuroprotective mechanism. Although our 16S data cannot confirm the species, this potential autoimmune link warrants urgent investigation using serological assays in PD cohorts.

Consistent with the “leaky gut” hypothesis, we observed a paradoxical increase in *Akkermansia* in the PDC group. While often regarded as a beneficial probiotic for metabolic health, recent reviews highlight its “double-edged sword” role in neurodegenerative diseases (Fang et al., 2021; Ilie et al., 2025). *Akkermansia* is an obligate mucin-degrader; in the context of constipation where transit time is prolonged, its excessive proliferation can lead to the over-degradation of the colonic mucus layer (Desai et al., 2016). This barrier erosion exposes the intestinal epithelium to bacterial endotoxins (LPS) and oxidative stress, thereby facilitating neuroinflammation and alpha-synuclein pathology in the enteric nervous system (Challis et al., 2020; Fang et al., 2021).

Functional prediction analysis further supported the link between dysbiosis and PD pathophysiology. At the KEGG Level 3 pathway level, the PDC group showed an overall trend toward reduced predicted functional abundance across several nominally significant pathways, suggesting broader metabolic impairment beyond taxonomic shifts alone. Among these, the depletion of tyrosine metabolism is particularly noteworthy, as tyrosine is a key precursor for dopamine synthesis and gut bacteria can influence levodopa bioavailability (Rekdal et al., 2019; van Kessel et al., 2019). Consistent with this interpretation, a 2025 study identified Gpr35 as a key regulator of the gut-brain axis and showed that its neuroprotective effects were associated with modulation of the tyrosine metabolism pathway (Meng et al., 2025). Likewise, the downregulation of glutathione metabolism suggests a weakened antioxidant defense capacity within the intestinal microenvironment, potentially increasing the susceptibility of enteric neurons to oxidative stress (Smeyne and Smeyne, 2013). In addition, the reduced abundance of xenobiotic degradation pathways, such as toluene and dioxin degradation, may reflect a diminished microbial capacity for environmental chemical processing. However, these findings should be interpreted cautiously, because PICRUSt provides inference-based functional predictions from 16S rRNA data rather than direct metagenomic or metabolomic measurements, and KEGG pathway labels do not necessarily indicate activation of the corresponding host biological pathways.

It is noteworthy that the beta-diversity analysis showed a significant but low-magnitude separation (R = 0.084) between PDC and FC. This high degree of structural overlap is expected, as colonic transit time is a dominant factor shaping the gut microbiota. Since both groups suffered from severe constipation, the global microbiome structure was likely homogenized by intestinal stasis. However, despite this shared ecological background, we detected robust differences in specific genera (e.g., *Paraprevotella*, *Akkermansia*), suggesting that these alterations are likely driven by PD-specific pathophysiology (e.g., systemic inflammation or autoimmunity) rather than motility issues alone.

Although this study effectively eliminated the interference of colonic transit time on gut microbiota by introducing functional constipation (FC) as a disease control, there are still several limitations. Firstly, this study is a single-center design, including only 35 subjects. The small sample size may weaken the statistical power to detect minor microbiota differences and limit the generalizability of the results. Secondly, the study did not include a healthy control group (HC), making it impossible to establish a baseline of microbiota under “healthy” conditions under the same experimental conditions. Lastly, as a cross-sectional case-control study, this study can only reveal associations at a specific time point and cannot establish a causal relationship between microbiota changes and PD progression; meanwhile, 16S rRNA sequencing has limited resolution at the strain level, and functional analysis is based on PICRUSt software prediction rather than direct metabolic measurements.

## 5 Conclusion

By employing functional constipation (FC) as a control for colonic transit time, this study identified specific gut microbial alterations associated with Parkinson’s disease, distinct from dysbiosis driven solely by motility issues. While the global community structure reflected the shared constipation phenotype, the PDC microbiome was characterized by specific compositional shifts, including the enrichment of *unclassified Ruminococcaceae*, the heterogeneous expansion of *Paraprevotella*, the increase of mucin-degrading *Akkermansia*, and the emergence of *Campylobacter*. These taxonomical changes, coupled with predicted deficits in tyrosine and glutathione metabolism, support a “gut-first” etiology involving inflammation and barrier compromise, highlighting these specific taxa as potential biomarkers independent of constipation severity.

## Data Availability

The data supporting the findings of this study are available from the corresponding author upon reasonable request.
